# The Sex Differences of Morphology and Immunology of SIRS of Newborn Wistar Rats

**DOI:** 10.1155/2014/190749

**Published:** 2014-11-06

**Authors:** A. M. Kosyreva

**Affiliations:** FSBI “SRI of Human Morphology” RAMS, Tsyurupa Street 3, Moscow 117418, Russia

## Abstract

The sex differences of infection and inflammatory diseases particularly appear at reproductive age and depend on the sex hormone level, varied between male and female. There are a few sets of data about the sex differences of infection and inflammatory diseases course, including systemic inflammatory response syndrome (SIRS) and sepsis, of newborns. The aim of our research was the estimation of morphological and immunological manifestation of SIRS of the newborn Wistar rats. Investigations were carried out on male and female two-day-old Wistar rats (10–12 g). SIRS was modeled by intraperitoneal injection of LPS (*E. coli*, O26: B6 strain, Sigma) in high dose—15 mg/kg. We did not find out any sex differences of the liver lesions severity between newborn males and females after LPS injection. The levels of endotoxin and estradiol in the serum, as the number of neutrophils in the intra-alveolar septa of the lungs, were higher in males than females with SIRS. Production of IL-2 and TNF-*α* by the spleen cells of males was higher than that in control group that reflects polarization predominantly on the Th1-type immune response. The secretion of IL-2, TNF-*α*, and IFN-*γ* by ConA activated spleen cells of females decreased that reflects the suppression of Th1-type immune response. We suppose that the LPS administration in the high dose causes the multidirectional reaction of the immune system of neonatal males and females Wistar rats.

## 1. Introduction

The sex differences of infection and inflammatory diseases particularly manifest at reproductive age. Many authors suggest it depends on the differences in the sex hormone level, varied between male and female [1]. According to Moore et al. [2], men are more frequently subjected to sepsis than females. Mortality from sepsis in adulthood is over 35% [3], when sepsis of a newborn occurs less frequently and child's mortality is not over 10% [4]. Shin et al. [5] have shown that neonatal sepsis is more common among boys. Unlike adult sepsis, clinical symptoms of neonatal sepsis are variable, minimal, and nonspecific [6, 7]. Innate immunity for neonate is more crucial than adaptive one because newborn “must” protect themselves from infection during the first days of life. In the large quantity of articles, it was suggested that the stages of innate immune mechanisms were impaired in infancy [8]. So the neonatal system under septic condition has a lower capacity to generate reactive oxygen and nitrogen species (ROS and RNS); different cytokines production compared to adulthood; low leukocyte subset numbers and an impaired capacity for migration, chemotaxis, endothelial adherence, and phagocytosis [7, 9–11]. However, there are a few sets of data about the sex differences of infection and inflammatory diseases course, including SIRS and sepsis, of newborns. Therefore, the aim of our research was the estimation of morphological and immunological manifestation of SIRS of the newborn Wistar rats.

## 2. Methods

### 2.1. Experimental Animals

Investigations were carried out on male (*n* = 25) and female (*n* = 21) two-day-old Wistar rats (10–12 g). The newborn Wistar rats were born of 15 adult animals (10 females and 5 males). Adult rats were housed in the polyacrylic cages (two females and one male per cage) and were maintained under the standard laboratory conditions (temperature: 20–22°C, relative humidity: 60–70%, and 12 h light/dark cycles). They were fed the commercial rat feed and boiled water ad libitum. The duration of pregnancy was 21–23 days. The offspring per female was presented by 6–8 individuals. The first 4 days after a rat's birth are neonatal period, characterized by high levels of estrogen in the blood serum [12]. The immune system of the newborn rats is in a state of physiological immunodeficiency, which is due to the functional immaturity of the immune cells [13]. All experiments involving animals were approved by the Animal Ethics Committee of “SRI of Human Morphology” RAMS.

### 2.2. Modeling of SIRS

Systemic inflammatory response syndrome (SIRS) was modeled by intraperitoneal injection of LPS (*E. coli*, O26:B6 strain, Sigma) in high dose—15 mg/kg. Earlier in preexperiment, it was shown that the use of the lower dose for the SIRS forming for adult Wistar rats (1.5 mg/kg) did not cause any morphological changes of SIRS in the target organs of newborn rats: we did not find cells injury and necrosis in the liver, intra-alveolar edema, and neutrophilic infiltration in the intra-alveolar septa of the lung. The injection of LPS to newborn rats in a higher dose, 15 mg/kg, resulted in morphological features of SIRS in the liver and the lung. The mortality rate of male rats was 50% and of females was not over 20%.

The rats of the control group were injected the same volume of saline solution intraperitoneally. Animals were killed by overdose of the diethyl ether on the 1st day after the LPS injection (females: *n* = 15, males: *n* = 15).

### 2.3. Morphology

We studied the liver and the lung. Exempt liver was fixated in Bouin's fluid, the lung was fixated in Carnoy's fluid, and organs were poured in paraffin. Histological sections were manufactured and stained by hematoxylin and eosin.

The number of neutrophils in the intra-alveolar septa of the lungs was counted in the histological slices in a standard field of view (25000 mkm^2^).

The severity of pathological changes at the liver was estimated by semiquantitatively double blind method in points according to the following scale: 0 points: the absence of any vacuolar degeneration; 0.5 points: less than 30% of hepatocytes with vacuolar degeneration; 1 point: 31–60% of hepatocytes with vacuolar degeneration; 2 points: more than 60% of hepatocytes with vacuolar degeneration; 3 points: 100% of hepatocytes with vacuolar degeneration; 4 points: hepatocytes with vacuolar degeneration and unit focal necrosis on the slice; 5 points: hepatocytes with vacuolar degeneration and widespread necrosis.

### 2.4. Isolation and Cultivation of Splenic Cells

In order to activate cytokines synthesis and secretion we cultivated 10^6^/mL spleen cells in 1 mL of culture medium with concanavalin A (5 mkg/mL) for 20 hours at 37°C and 5% CO_2_ in 24-well cultured plates. The culture medium consists of RPMI-1640 (PanEco, Russia), 5% of inactivated fetal calf serum, 2 mM of glutamine, and 50 mkg/mL of gentamicin.

### 2.5. ELISA

Venous blood from jugular veins was centrifuged for 20 minutes at 200 g. The obtained serum was frozen at −70°C and stored no more than 2 months. We estimated the concentration of corticosterone and neopterin (IBL, Germany), total testosterone (DBC, Canada), estradiol (Cusabio, China) and progesterone (DBC, Canada), and TGF-*β* (eBioscience, Austria) in the serum by ELISA. The endotoxin level in the serum was estimated by chromogenic LAL-test (HBT, USA). In the cultured fluid of splenic cells, we studied the concentration of IL-2, IL-4, TNF-*β*, and IFN-*γ* by ELISA test systems of “eBioscience” (Austria).

### 2.6. Biochemical Methods

In order to estimate the severity of liver pathological changes in SIRS, we determined the activity of indicator enzymes, aspartate transaminase (AST) and alanine transaminase (ALT) [Enzyme Classification (EC) 2.6.1.1]. We used the “DiaSys” test systems (Diagnostic Systems Gmbh, Germany).

### 2.7. Statistics

Digital data were tested for normality using the Kolmogorov-Smirnov test in the program Statistica 7.0. The comparison of the normally distributed data was made using the parametric Student *t*-test, and when this was not the case we used the nonparametric Mann-Whitney *U* test. The median and interquartile range (Med: 25%–75%) or the arithmetic mean and standard error of the mean (M ± SE) were calculated for values of the measured parameters. The differences were considered statistically significant when *P* < 0.05.

## 3. Results

### 3.1. The Sex Differences of Sex Steroids' and Cytokines' Levels of Newborn Wistar Rats

The level of testosterone, progesterone, and corticosterone in the serum of newborn male Wistar rats did not differ from the level of newborn female Wistar rats. We found differences between male and female only in estradiol level, where female parameters were higher (Table 1).

Analyzing the cytokines production of newborn Wistar rats, we discovered that female culture of spleen cells had higher concentration of Th1-cytokines, IL-2 and IFN-*γ*, and proinflammatory cytokine, TNF-*α*, than male one (Table 2).

### 3.2. The Sex Differences of Morphological and Immunological Features of SIRS of Newborn Wistar Rats

#### 3.2.1. Steroid and Endotoxin Level in the Blood Serum

On the 1st day after the LPS administration, the level of endotoxin in the blood serum of 2-day-old male and female Wistar rats increased. However, compared to males, females had more pronounced increase of endotoxin concentration (Table 1). The levels of male's and female's corticosterone, testosterone, and progesterone did not differ from the control group in these terms of SIRS development (Table 1). Both females' and males' estradiol levels in the serum decreased; however, statistically significant differences in this index showed only males (Table 1).

#### 3.2.2. Pathological Changes in the Liver

**Figure 1:**
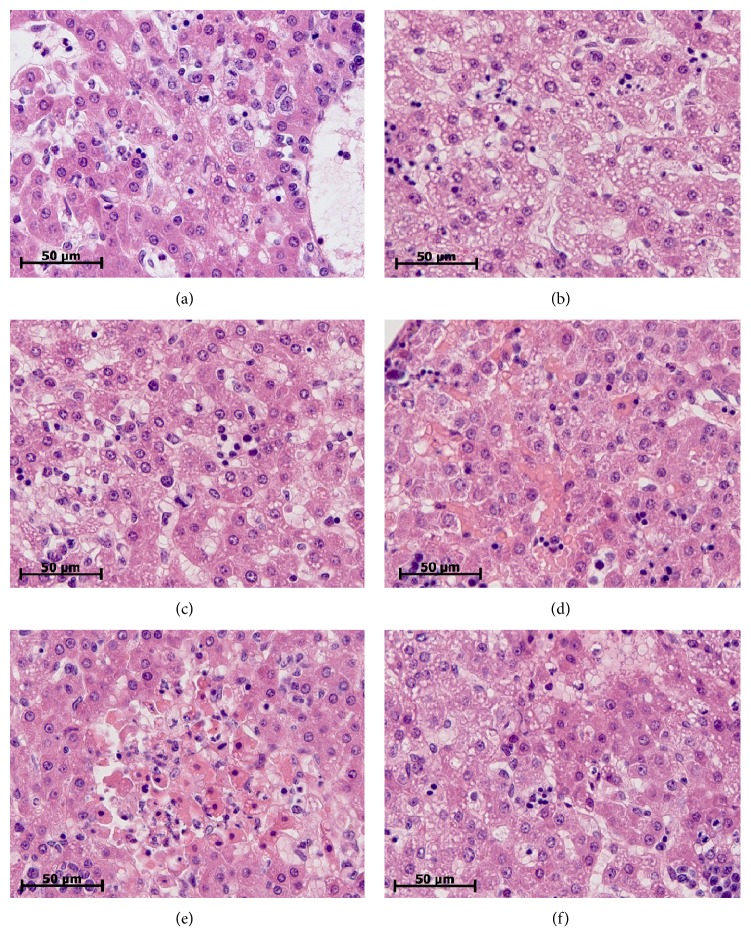
Variability of pathologic changes of the liver of 2-day-old rats of both sexes on the 1st day after LPS injection. (a) Mild dystrophic changes of hepatocytes (male). (b) Severe dystrophic changes of hepatocytes (female). (c) Mild dystrophic changes of hepatocytes (female). (d) Vascular congestion, stasis, and sludge (male). (e) Landscape necrosis (female). (f) Necrosis and severe dystrophic changes of hepatocytes (male). Hematoxylin and eosin staining. ×640.

**Figure 2:**
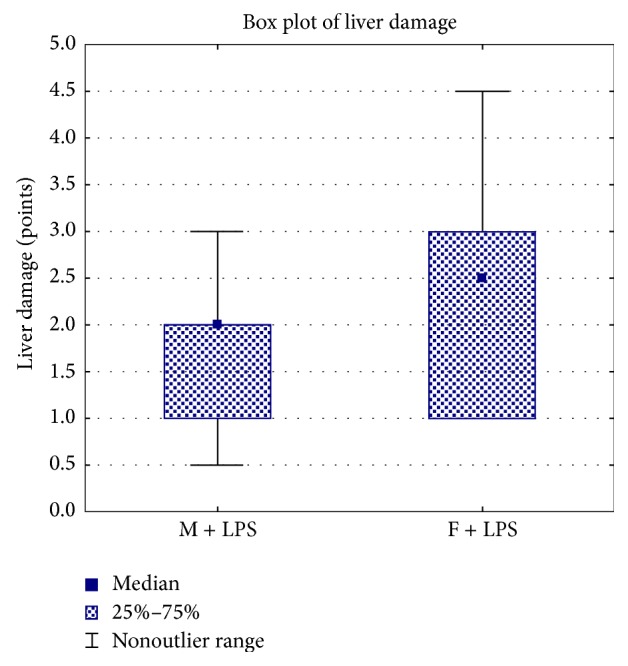
The semiquantitative assessment of the liver lesions severity of newborn male (M) and female (F) Wistar rats on the 1st day after LPS injection.

Morphologic study of the liver of males and females revealed specific pathological changes for SIRS. Vacuolar degeneration of hepatocytes was different inside groups and varied from mild to severe and total pathological changes (Figures 1(a)-1(c)). In the single events we found out focal necrosis (Figures 1(e)–1(f)). Both male and female rats had vascular congestion, stasis and sladge in the blood vessels of the liver (Figure 1(d)). We detected a fibrin in the lumen of the blood vessels. Extensive landscape necrosis and hemorrhage were found in the liver of the one from 10 males. The semiquantitative assessment of the liver lesions severity did not show any sex differences between newborn males and females. The median and the interquartile ranges of pathological changes' severity of hepatocytes of males were 2 (1-2) points, while those of females were −2.5 (1–3) points (Figure 2).

Biochemical analysis of liver enzymes showed that on the 1st day after LPS administration both male's and female's ALT activity level significantly increased (Table 1). However, in these periods of the experiment male and female newborn Wistar rats' level of AST activity did not change (Table 1).

#### 3.2.3. Pathological Changes in the Lung

**Figure 3:**
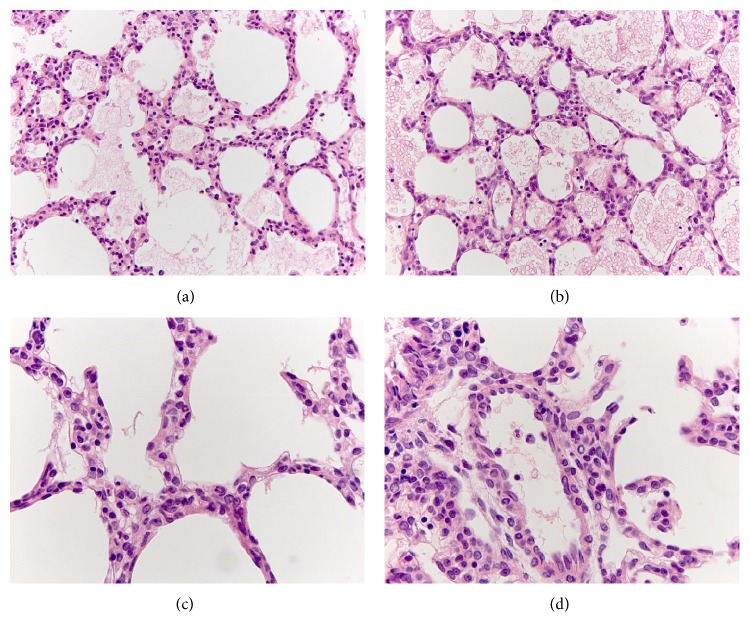
Morphologic changes in the lung of female ((a), (c)) and male ((b), (d)) 2-day-old rats on the 1st day after LPS injection. Vast areas of alveolar edema and gaps filled with alveolar erythrocytes and edematous liquid ((a), (b)). Diffusely scattered polymorphonuclear leukocytes in intra-alveolar septa around blood vessels ((c), (d)). Hematoxylin and eosin staining. (a), (b) ×320; (c), (d) ×640.

**Figure 4:**
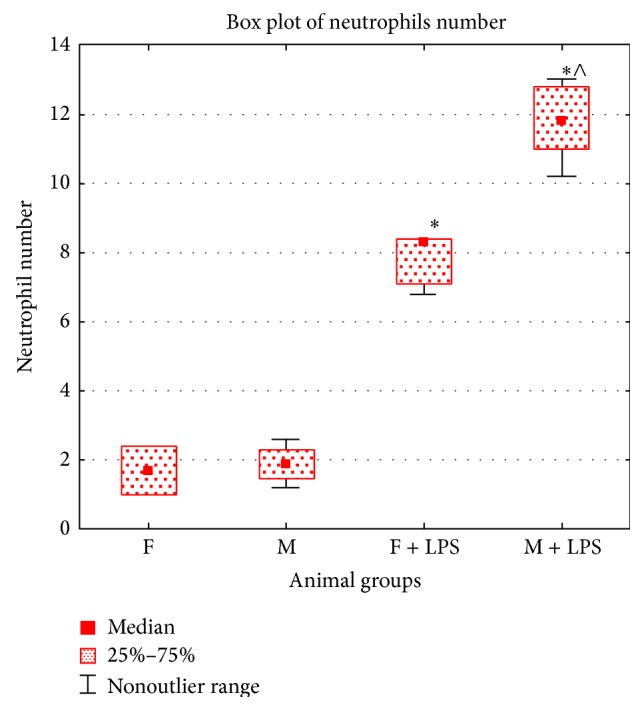
Neutrophil number in intra-alveolar septa of the lung of newborn male (M) and female (F) Wistar rats in two control groups (F, M) and on the 1st day after LPS injection (F + LPS, M + LPS). Symbols: ∗ means that differences are statistically significant as compared with the corresponding control group; ∧ means that differences are statistically significant as compared with the LPS injected females.

On the 1st day after LPS injection, vast areas of alveolar edema were detected in the lungs of newborn males and females. The alveolar gaps were filled with erythrocytes and homogeneous eosinophilic masses (Figures 3(a) and 3(b)). The number of neutrophils in the intra-alveolar septa of the lung increased (Figures 3(c) and 3(d)). Compared with females, the number of male neutrophils was higher in this period of SIRS (Figure 4) that reflects the greater severity of the inflammatory response in the male lung.

#### 3.2.4. Production of Cytokines

After the LPS administration, different dynamics of cytokine secretion by spleen cells of male and female Wistar rats is observed. Thus, females showed reduction in the level of IL-2 and TNF-*α*, whereas males showed the increase of production of these cytokines (Table 2). However, on the 1st day of SIRS production of IFN-*γ* in the culture fluid of spleen cells of males and females was decreased (Table 2). The concentration of neopterin increased in the females' serum, whereas, in males' serum, this index did not differ from the control group (Table 2). TGF-*β* level in the blood serum of the experimental groups did not differ from control ones (Table 2).

## 4. Discussion

Thus, compared with males, serum estradiol level and the production of IL-2, IFN-*γ*, and TNF-*α* of newborn female Wistar rats of the control group were higher. According to Konkle and McCarthy [14], on the 1st day after birth the level of estradiol in the serum of newborn male and female Sprague-Dawley rats is high and its concentration gradually declines after 48 hours of postnatal ontogenesis. In authors' opinion, high concentrations of estradiol have their maternal origin and their further reduction is associated with newborn rats' metabolic processes. According to our data, the 2-day-old female Wistar rats have higher estradiol level than males that is apparently due to less active metabolism of estrogen of female Wistar rats.

It has been shown that newborn babies have higher number of T cells producing IL-2, and lower rate of T cells producing IFN-*γ* than older children [15]. Wiegering et al. [16] have found that the IFN-*γ* and TNF-*α* production levels by T-helper cell increase with age. In addition, the authors found that infant boys have higher level of IL-2 production by lymphocytes than girls. We have, however, been shown that the level of Th1 cytokines production, mediating the cellular immune response of the newborn female Wistar rats, is higher than male response. It could be associated with species' characteristics or with the activation of the innate immune reactions due to the immunostimulatory effect of estrogens. According to the literature, monocytes of neonates are more sensitive to estradiol and progesterone than ones of adults that are associated with the high expression of receptors for the female sex steroids on infants' monocytes [17].

On the 1st day after LPS administration, the development of SIRS of newborn males and females was accompanied by the increase of endotoxin level and ALT activity level in serum, alterative and inflammatory changes in the liver and lungs, and changes in the level of cytokine production. According to the literature, the particularity of clinical manifestations and changes in laboratory parameters of infants characterizing the neonatal sepsis is associated primarily with the functional immaturity of the immune system. Compared to adults, neonates have lower cytokine production and bactericidal activity of phagocytes, and, moreover, there is a low activity of the antigen-presenting cells, T-lymphocytes, and immunoglobulin synthesis in children of the 1st month of their life [18–20]. Giannoni et al. [17] have shown that the expression level of toll-like receptors (TLR2 and TLR4) on monocytes of a newborn is below than in adults. This may be one reason why we found less pronounced sex differences and alterative degenerative changes in the liver of neonatal Wistar rats with SIRS compared with mature animals [21].

On the 1st day of the development of SIRS, estradiol and endotoxin concentrations in the serum and the number of neutrophils in the intra-alveolar septa of the females' lungs were higher than male results. In this period in the culture liquid of male spleen cells, production of IL-2 and TNF-*α* increases as compared with the control group that reflects the polarization mainly of Th1-type of the immune response. According to the literature, estradiol binding to nuclear receptors has anti-inflammatory effects by inhibiting NF-*κ*B-dependent cascade of intracellular reactions, thereby reducing the level of production of proinflammatory cytokines, TNF-*α* and IL-6 [17]. We found an increase of IL-2 and TNF-*α* production levels in neonatal male spleen cell culture, which correlated with the low levels of estradiol in the serum in the 1st day of SIRS development. Females, on the contrary, showed higher level of estradiol than males, which was accompanied by a decrease in production of IL-2, TNF-*α*, and IFN-*γ*. More pronounced polarization of the immune response to Th1-type of males explains the high number of neutrophils in the lung intra-alveolar septa compared with females whose neutrophilic infiltration was less pronounced.

Thus, morphological and immunological manifestation of SIRS of newborn male and female Wistar rats has several features. As compared with males, females have a more pronounced increase of endotoxin level in the blood serum. As for males, it is vice versa; there is a marked reduction of estradiol levels in serum. According to analysis of cytokine profile neonatal female Wistar rats have reduced levels of IL-2, IFN-*γ*, and TNF-*α*, while males have increased IL-2 and TNF-*α* secretion, which indicates that the reaction of the immune system of neonatal males and females is multidirectional after the LPS administration in the high dose.

## 5. Conclusions

Two-day-old female Wistar rats of control group showed higher serum estradiol level and production of IL-2, IFN-*γ*, and TNF-*α* in spleen cells culture than males. The development of SIRS of male and female newborns is accompanied by the increase of endotoxin level and ALT activity in serum, alterative and inflammatory changes in the liver and lungs, and changes in the level of cytokine production. The levels of endotoxin and estradiol in the serum, as the number of neutrophils in the intra-alveolar septa of the lungs, were higher in males than in females with SIRS. Production of IL-2 and TNF-*α* by the spleen cells of males was higher than that in control group, which reflects polarization predominantly on the Th1-type immune response. On the 1st day after LPS administration females showed a reduction in the secretion of IL-2, TNF-*α*, and IFN-*γ* by ConA activated spleen cells that reflects the suppression of Th1-type immune response.

## Figures and Tables

**Table 1:** Endotoxin, corticosterone, and sex hormones levels in the serum of 2-day-old male and female Wistar rats in two control groups and on the 1st day after LPS injection (Med: 25%–75%).

	Females		Males	
	Control group	LPS 1	Control group	LPS 1
Endotoxin (eU/ml)	1.9 (0.5–3.3)	57^*^ (47.3–90.7)	0.5 (0.1–2.6)	33.5^∗&^ (29.7–44.3)
Corticosterone (nmol/l)	122.3 (112–440)	436.3 (235.5–447.5)	215.1 (35.9–340)	351.3 (210.6–460)
Total testosterone (ng/ml)	0.2 (0.2-0.3)	0.4 (0.2–0.5)	0.3 (0.1–3.3)	1.7 (1.5–2.1)
Estradiol (pg/ml)	32.9 (32–34.5)	19.5 (13.2–21.8)	22.2^#^ (20.2–24.6)	8.4^*^ (6.7–21.5)
Progesterone (ng/ml)	1.2 (0.6–1.2)	0.5 (0.2–0.8)	0.8 (0.7-0.8)	0.5 (0.3–0.8)
ALT (eU/l)	22 (13–41)	107^*^ (89–222)	50.5 (29–74)	102^*^ (52–174)
AST (eU/l)	264 (217–422)	512 (244–685)	197 (132.5–371.5)	294 (179–343)

Med: median, 25%–75%: interquartile range, LPS 1: the 1st day after LPS injection, ^*^differences which are statistically significant as compared with the corresponding control group, ^&^differences which are statistically significant between males and females after LPS injection, and ^#^differences which are statistically significant between control groups of males and females.

**Table 2:** Production level of cytokines by ConA activated splenic cells and neopterin and TGF-*β* concentration in the serum of 2-day-old males and females in two control groups and on the 1st day after LPS injection (Med: 25%–75%).

	Females		Males	
	Control group	LPS 1	Control group	LPS 1
IL-2 (pg/ml)	4403 (2378–6093)	456.3^*^ (386.1–617.8)	141.7^#^ (124.6–158.8)	474.4^*^ (426.1–826.2)
IL-4 (pg/ml)	0.2 (0–0.9)	0.4 (0.1–0.8)	0.1 (0–0.3)	0.3 (0–0.6)
IFN-*γ* (pg/ml)	48.9 (32.2–57.9)	0^*^ (0-0)	0.9^#^ (0.3–10.9)	0^*^ (0-0)
TNF-*α* (pg/ml)	169.7 (123.6–255.8)	89.8^*^ (67.4–128.8)	10.8^#^ (1.3–13.5)	51.2^*^ (41.8–70.1)
Neopterin (нмольл)	7 (5.7–10.8)	14.2^*^ (11.6–14.2)	9.1 (7.1–10.4)	7.3 (6–8.7)
TGF-*β* (ng/ml)	18.6 (16.1–20.9)	14.5 (15.2–18.2)	18.9 (16.8–27.1)	17 (15.2–18.2)

Med: median, 25%–75%: interquartile range, LPS 1: the 1st day after LPS injection, ^*^differences which are statistically significant as compared with the corresponding control group, and ^#^differences which are statistically significant between control groups of males and females.
